# Supplementary material for: Vaccination with mRNA-encoded membrane-bound HIV Envelope trimer induces neutralizing antibodies in animal models
Source: bioRxiv. 2025 Jan 25:2025.01.24.634423. Preprint. [Version 1] doi: 10.1101/2025.01.24.634423 (PMC11785158; doi:10.1101/2025.01.24.634423)
Supplement: Supplement 1 — Fig. S1. Characterization of BG505 MD39 soluble immunogens. (A) Biolayer interferometry (BLI) was used to assess antigenic profiles for the indicated trimers binding to IgGs for bnAbs (quaternary, PGT151 and PGT145; CD4bs, VRC01; and V3-glycan, PGT121 and PGT128) and non-nAbs (V3, 19b; CD4bs, B6 and F105). (B) Yield and thermostability of BG505 MD39 based immunogens. Yield was determined after 2G12 affinity chromatography and SEC purification. Thermostability measurements were made using nano differential scanning fluorimetry. (C) Negative stain electron microscopy analysis of BG505 MD39.3 gp140. (D) Glycan analysis of BG505 MD39 gp140 and BG505 MD39.3 gp140. Green indicates high mannose glycans, pink indicates complex type glycans and gray indicates unoccupied glycosylation sites. The N241 and N289 glycosylation sites are not present on BG505 MD39 gp140. Glycosylation sites are numbered using HxB2 numbering. *Adapted with permission from (47). (E) Negative stain electron microscopy analysis of BG505 SOSIP gp140 or BG505 MD39 gp140 with human sCD4. (F) Binding of sCD4 followed by 17b IgG for BG505 SOSIP, BG505 MD39, BG505 MD39.3, and BG505 MD39.3-CD4KO assessed by BLI. Fig. S2. Cell surface antigenicity. Flow cytometry analysis of HEK293F cells transfected with DNA plasmids encoding membrane-bound HIV Env constructs. Cells were stained with bnAbs quaternary specific (PGT145 and PGT151), CD4bs (VRC01), V3-glycan (PGT121 and PGT128), MPER (10E8), or non-neutralizing antibodies (F105, B6, and 19b). (A) Raw MFI values show expression levels of each construct. Median values plotted with error bars showing the range (n=2). (B) PGT121 normalized MFI values showing antigenicity scaled to expression levels for each construct. (C) Comparison of effect of glycan hole for autologous neutralization. Week 26 serum neutralization against BG505 T332N pseudovirus of MD39.3 gp140 protein (G5) with glycan hole-containing MD39 gp140 protein NHP animals used in Silva et al (20). Bars in [file media-1.pdf]

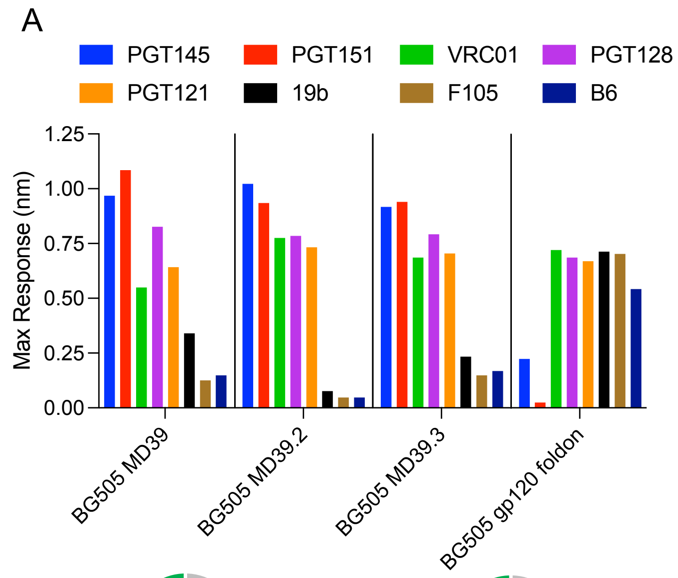

B

Name	Post SEC Yield (mg/L)	Tm (°C)
BG505 MD39 gp140	3.6	78.7
BG505 MD39.2 gp140	3.3	73.6
BG505 MD39.3 gp140	3.1	75.6

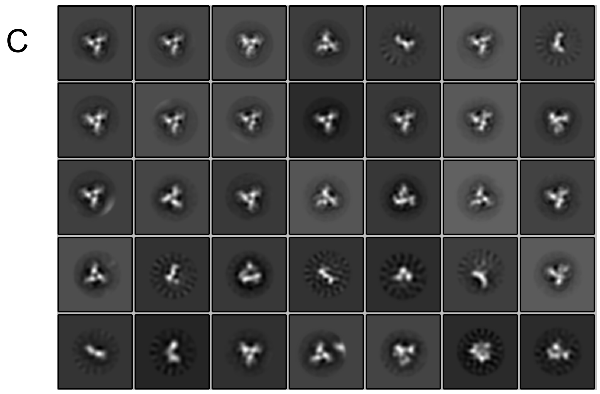

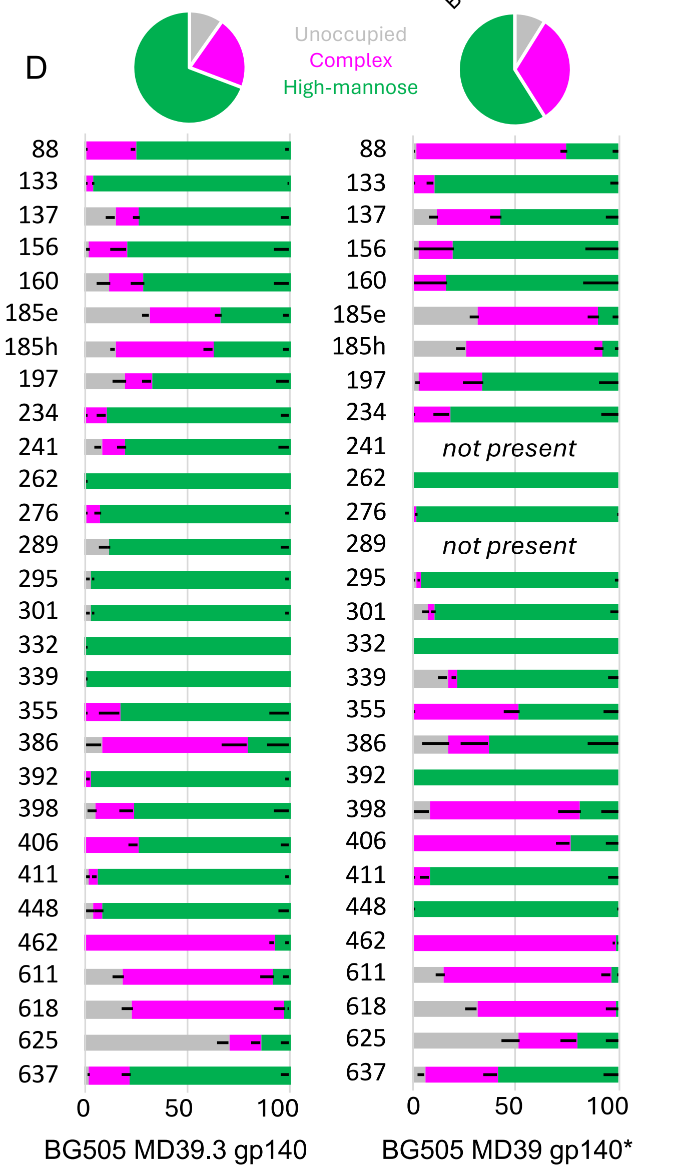

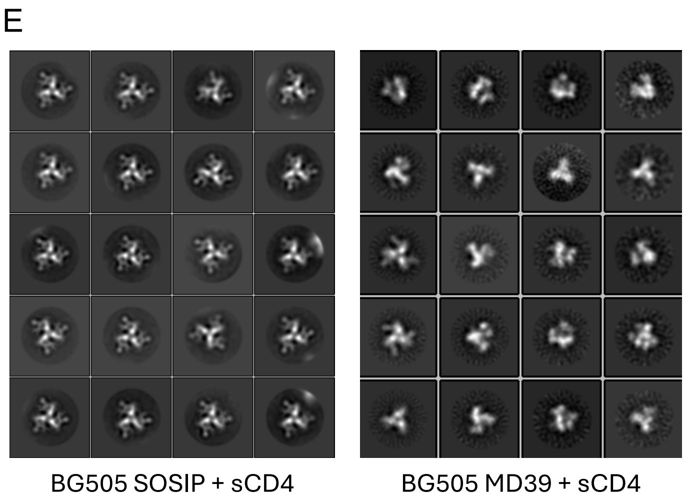

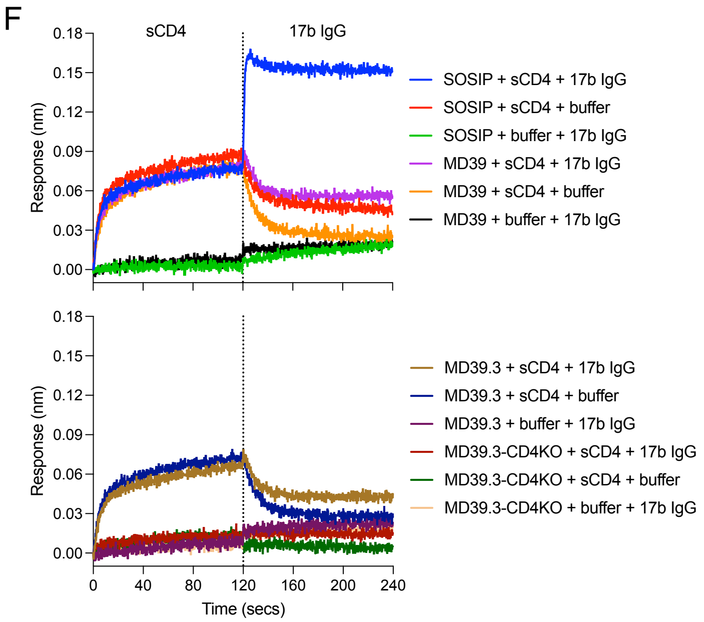

Figure S1

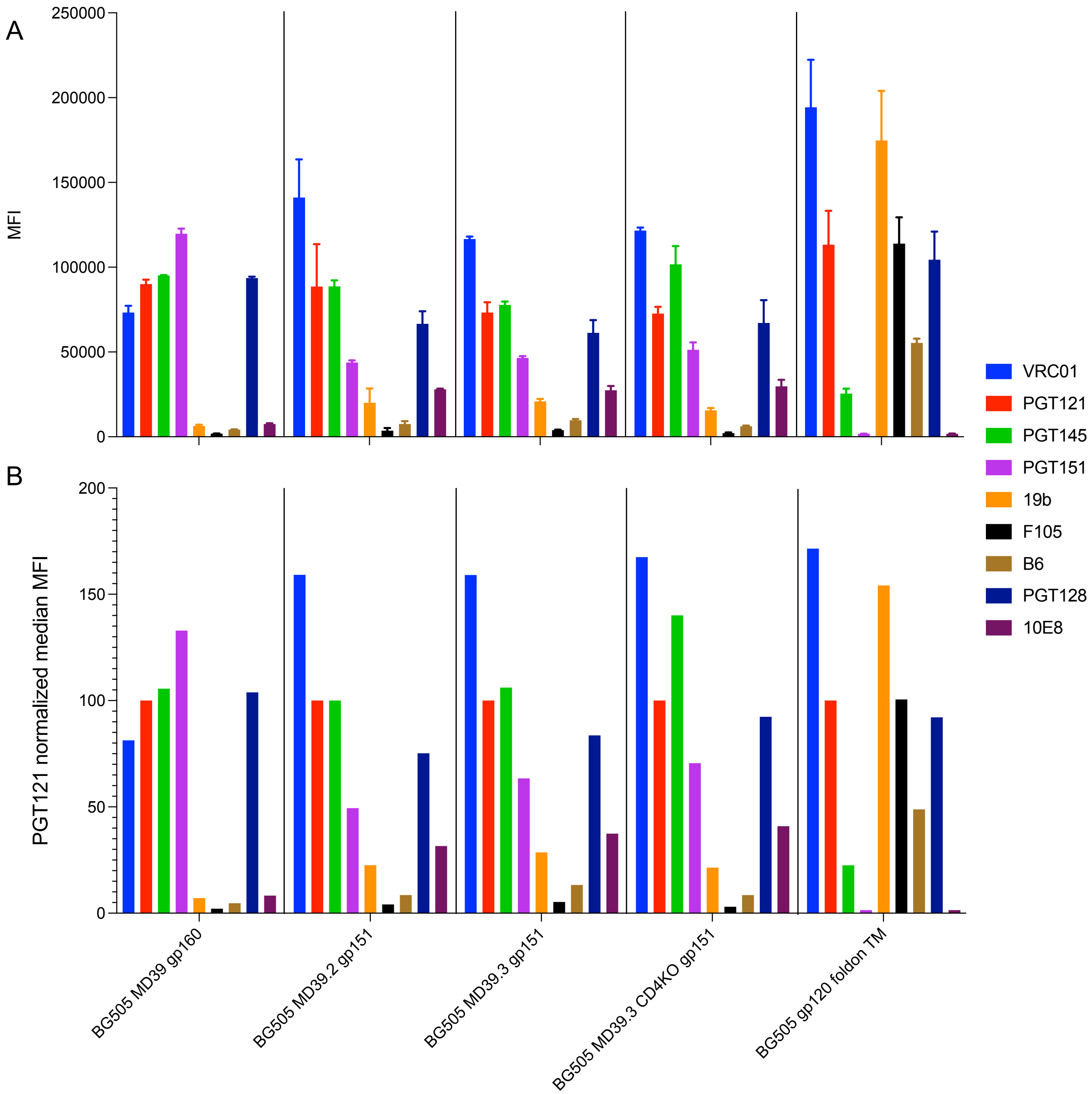

C

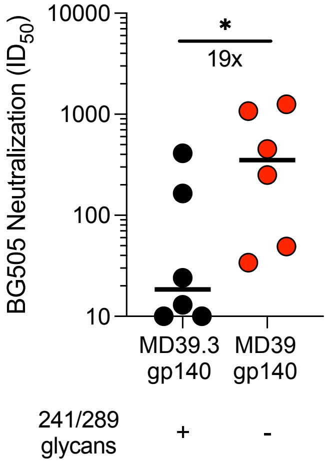

group	animal	ID50	geo mean	median
MD39.3 gp140	RBa19	13	36	19
	REy18	165		
	RWh18	10		
	RWm18	410		
	RWn18	24		
	RYt17	10		
MD39 gp140	RJc18	251	252	353
	RMs17	34		
	RGu17	454		
	34880	49		
	RNv17	1252		
	RUs17	1076		

Figure S2

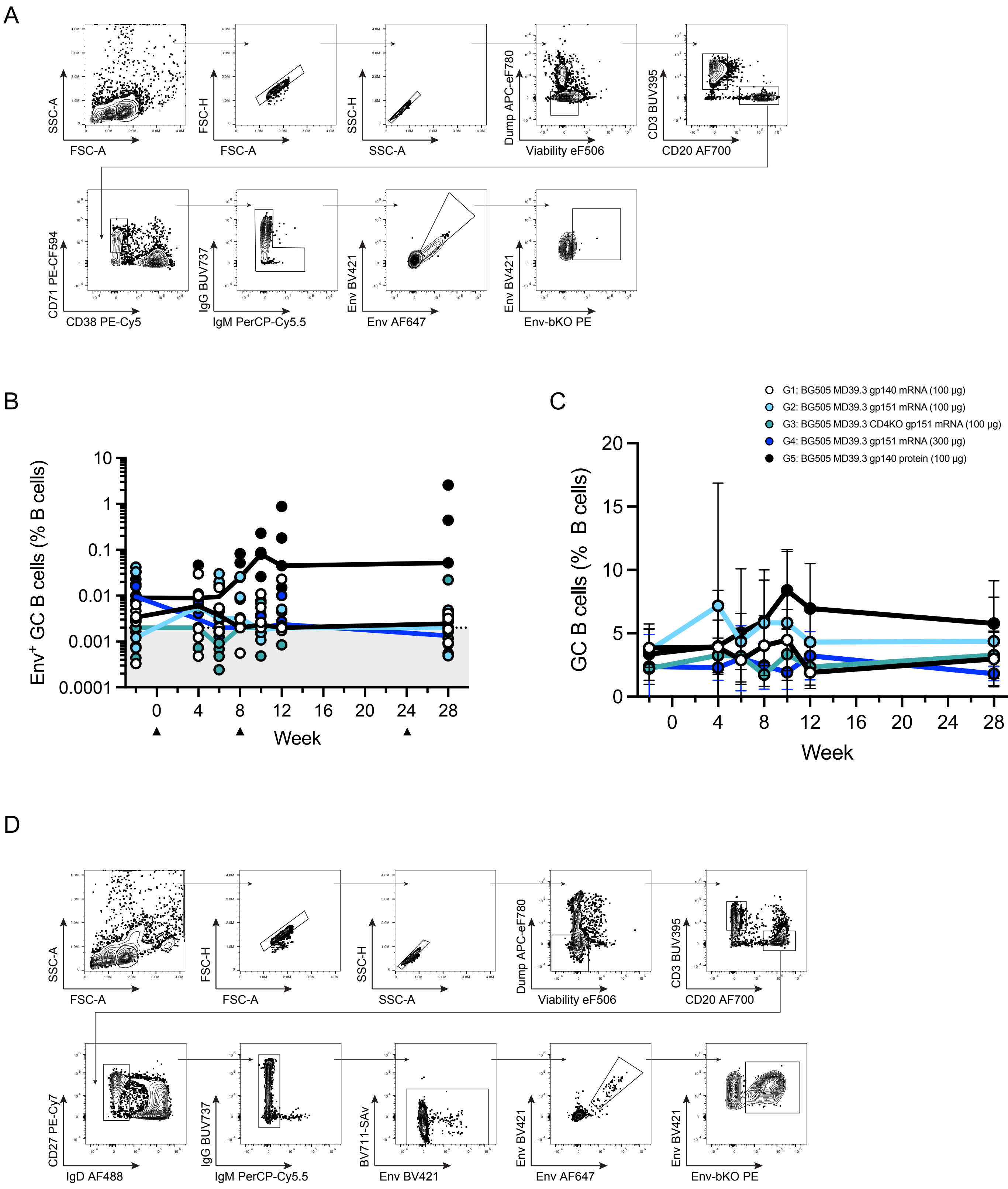
A
SSC-A
FSC-A
FSC-H
FSC-A
SSC-H
SSC-A
Dump APC-eF780
Viability eF506
CD3 BUV395
CD20 AF700
CD71 PE-CF594
CD38 PE-Cy5
IgG BUV737
IgM PerCP-Cy5.5
Env BV421
Env AF647
Env BV421
Env-bKO PE
B
Env+ GC B cells (% B cells)
10
1
0.1
0.01
0.001
0.0001
0
4
8
12
16
20
24
28
Week
C
G1: BG505 MD39.3 gp140 mRNA (100 µg)
G2: BG505 MD39.3 gp151 mRNA (100 µg)
G3: BG505 MD39.3 CD4KO gp151 mRNA (100 µg)
G4: BG505 MD39.3 gp151 mRNA (300 µg)
G5: BG505 MD39.3 gp140 protein (100 µg)
GC B cells (% B cells)
20
15
10
5
0
0
4
8
12
16
20
24
28
Week
D
SSC-A
FSC-A
FSC-H
FSC-A
SSC-H
SSC-A
Dump APC-eF780
Viability eF506
CD3 BUV395
CD20 AF700
CD27 PE-Cy7
IgD AF488
IgG BUV737
IgM PerCP-Cy5.5
BV711-SAv
Env BV421
Env BV421
Env AF647
Env BV421
Env-bKO PE

Figure S3

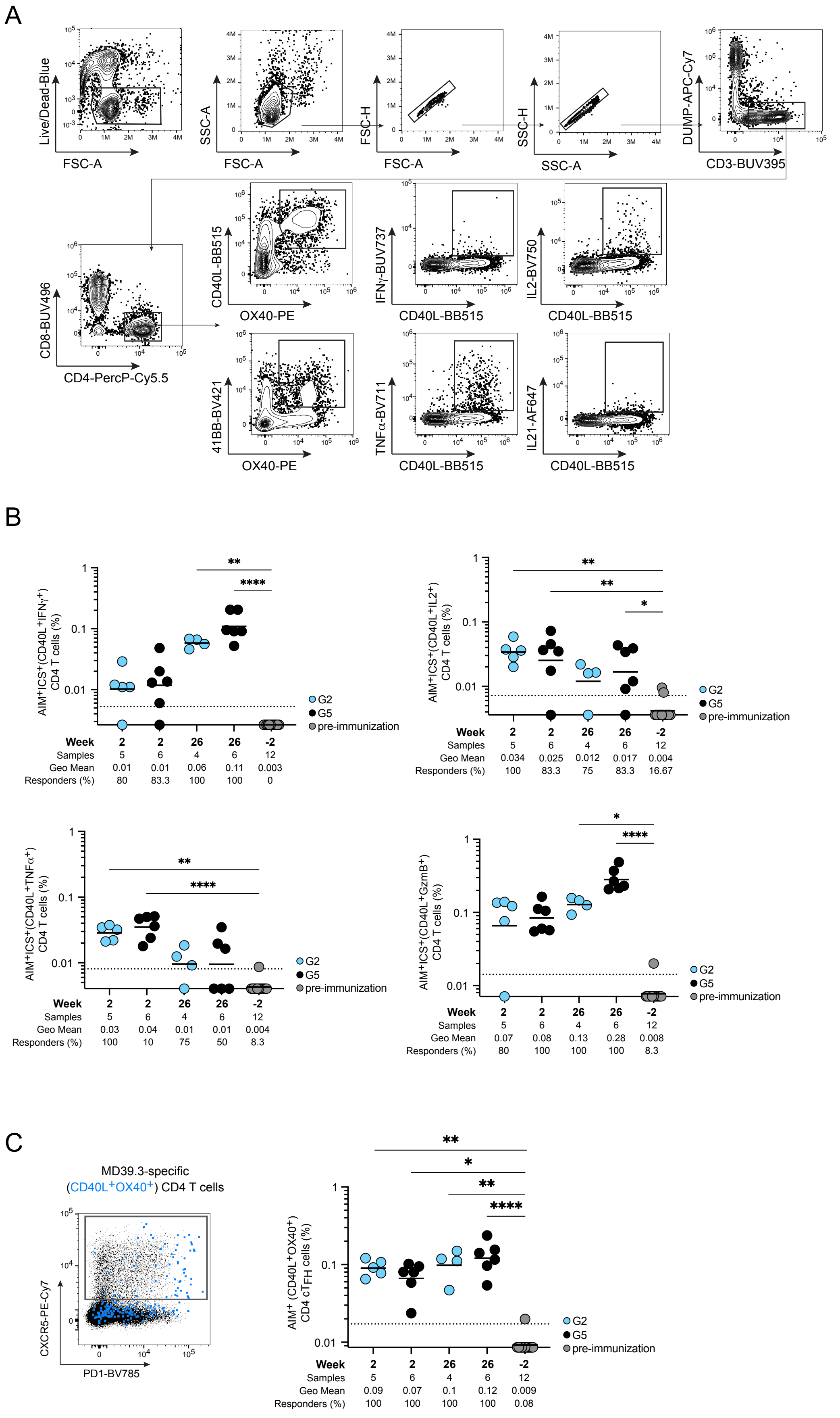
A
Live/Dead-Blue
FSC-A
SSC-A
FSC-A
FSC-H
FSC-A
SSC-H
SSC-A
DUMP-APC-Cy7
CD3-BUV395
CD8-BUV496
CD4-PercP-Cy5.5
CD40L-BB515
OX40-PE
IFNγ-BUV737
CD40L-BB515
IL2-BV750
CD40L-BB515
41BB-BV421
OX40-PE
TNFα-BV711
CD40L-BB515
IL21-AF647
CD40L-BB515
B
AIM+ICS+(CD40L+IFNγ+) CD4 T cells (%)
G2
G5
pre-immunization
Week 2 2 26 26 -2
Samples 5 6 4 6 12
Geo Mean 0.01 0.01 0.06 0.11 0.003
Responders (%) 80 83.3 100 100 0
AIM+ICS+(CD40L+IL2+) CD4 T cells (%)
G2
G5
pre-immunization
Week 2 2 26 26 -2
Samples 5 6 4 6 12
Geo Mean 0.034 0.025 0.012 0.017 0.004
Responders (%) 100 83.3 75 83.3 16.67
AIM+ICS+(CD40L+TNFα+) CD4 T cells (%)
G2
G5
pre-immunization
Week 2 2 26 26 -2
Samples 5 6 4 6 12
Geo Mean 0.03 0.04 0.01 0.01 0.004
Responders (%) 100 10 75 50 8.3
AIM+ICS+(CD40L+GzmB+) CD4 T cells (%)
G2
G5
pre-immunization
Week 2 2 26 26 -2
Samples 5 6 4 6 12
Geo Mean 0.07 0.08 0.13 0.28 0.008
Responders (%) 80 100 100 100 8.3
C
MD39.3-specific
(CD40L+OX40+) CD4 T cells
CXCR5-PE-Cy7
PD1-BV785
AIM+ (CD40L+OX40+) CD4 cTFH cells (%)
G2
G5
pre-immunization
Week 2 2 26 26 -2
Samples 5 6 4 6 12
Geo Mean 0.09 0.07 0.1 0.12 0.009
Responders (%) 100 100 100 100 0.08

Figure S4

A

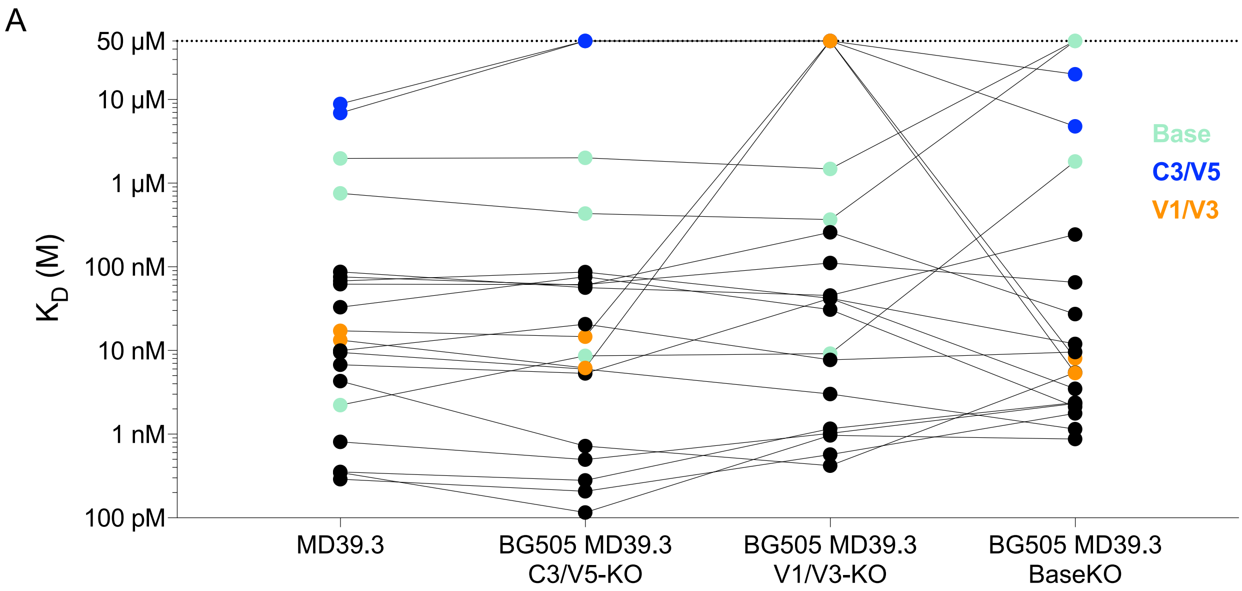

B

	BG505 WT	BG505 T332N	BG505 N611A	BG505 N241+N289	BG505 133aN	BG505 133aN+136aA	BG505 T465N	MLV
VRC01	0.084	0.047	0.089	0.050	0.120	0.091	0.084	NN
RFu18_1_IgG	NN	NN	NN	NN	NN	NN	NN	NN
RFu18_10_IgG	NN	NN	NN	NN	NN	NN	NN	NN
RFu18_12_IgG	NN	NN	NN	NN	NN	NN	NN	NN
RFu18_2_IgG	NN	NN	NN	NN	NN	NN	NN	NN
RFu18_4_IgG	NN	NN	NN	NN	NN	NN	NN	NN
RFu18_6_IgG	NN	NN	NN	NN	NN	NN	NN	NN
RFu18_37_IgG	NN	NN	NN	NN	NN	NN	NN	NN
RFu18_38_IgG	NN	NN	NN	NN	NN	NN	NN	NN
RUv18_29_IgG	NN	NN	NN	NN	NN	NN	NN	NN
RUv18_30_IgG	NN	NN	NN	NN	NN	NN	NN	NN
RUv18_31_IgG	NN	NN	NN	NN	NN	NN	NN	NN
RUv18_40_IgG	NN	NN	NN	NN	NN	NN	NN	NN
RUv18_42_IgG	NN	NN	NN	NN	NN	NN	NN	NN
RUv18_43_IgG	1.477	4.154	1.072	12.911	NN	NN	13.557	NN
RFu18_11_IgG	NN	21.955	NN	NN	NN	NN	NN	NN
RUv18_44_IgG	0.094	0.549	0.360	0.730	NN	NN	0.960	NN
RUv18_46_IgG	NN	NN	NN	NN	NN	NN	NN	NN
RUv18_47_IgG	NN	NN	NN	NN	NN	NN	NN	NN
RWo17_32_IgG	NN	NN	NN	NN	NN	NN	NN	NN
DEN	NN	NN	NN	NN	NN	NN	NN	NN

IC_{50} (μg/mL)
0.001-0.010
0.010-0.100
0.100-1.000
1.000-10.00
10.00-50.00

C

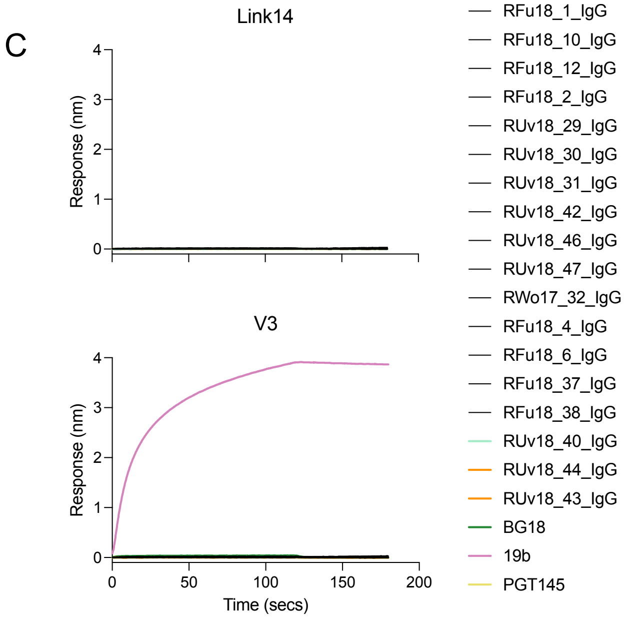

Figure S5

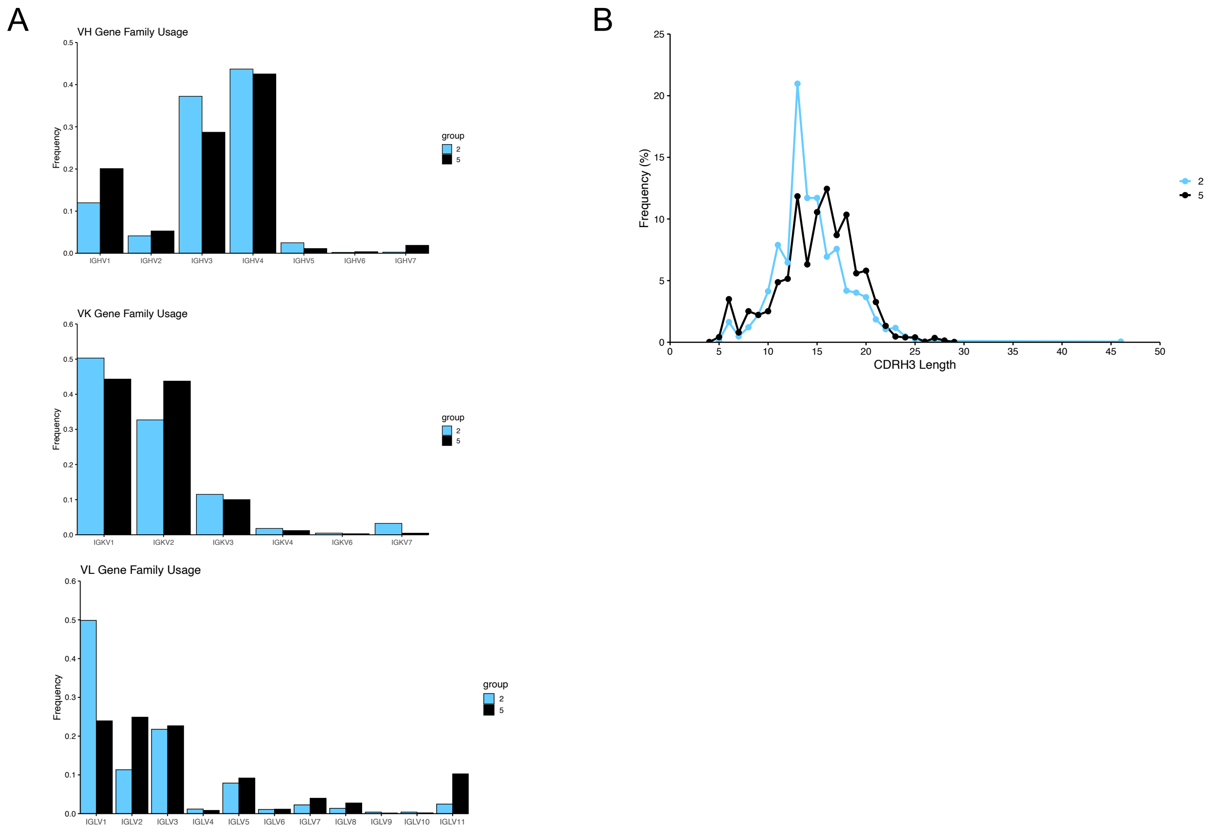
A
VH Gene Family Usage
Frequency
group
2
5
IGHV1
IGHV2
IGHV3
IGHV4
IGHV5
IGHV6
IGHV7
VK Gene Family Usage
IGKV1
IGKV2
IGKV3
IGKV4
IGKV6
IGKV7
VL Gene Family Usage
IGLV1
IGLV2
IGLV3
IGLV4
IGLV5
IGLV6
IGLV7
IGLV8
IGLV9
IGLV10
IGLV11
B
Frequency (%)
CDRH3 Length

Figure S6

A

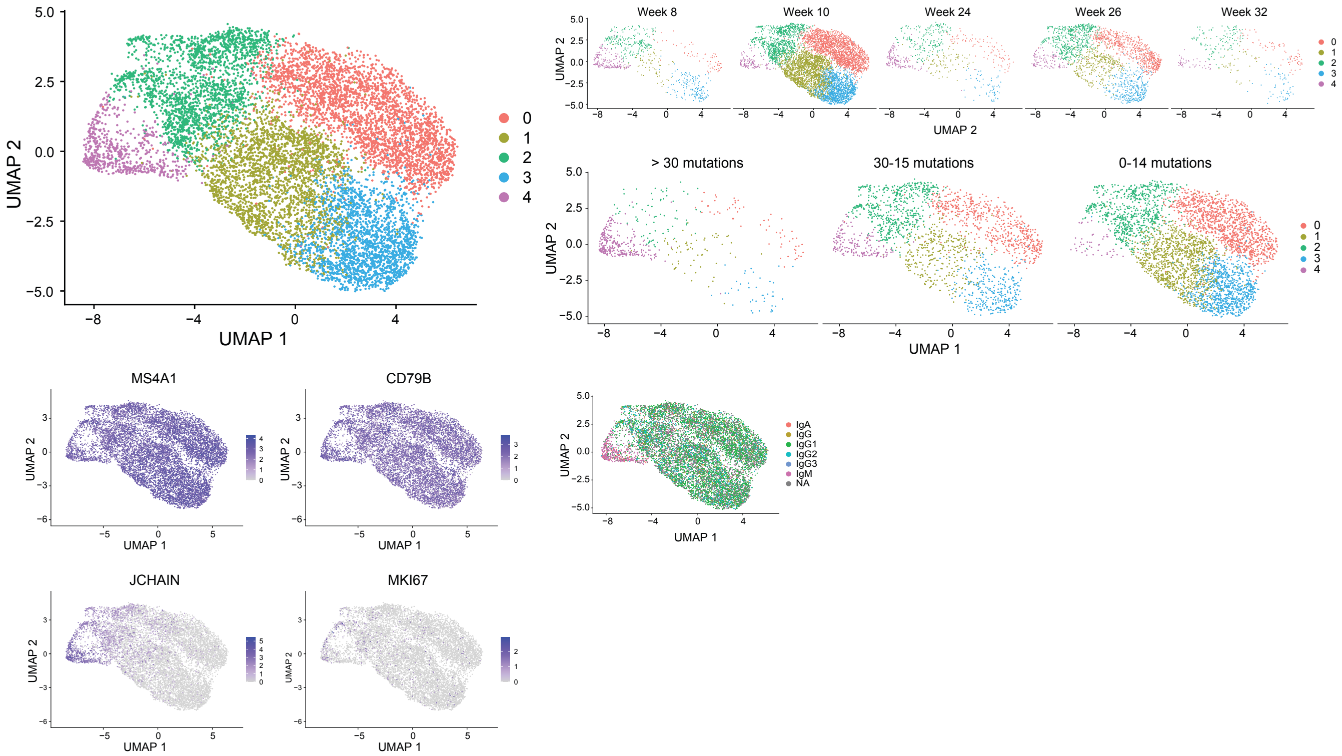

UMAP 1
UMAP 2
Week 8
Week 10
Week 24
Week 26
Week 32
> 30 mutations
30-15 mutations
0-14 mutations
MS4A1
CD79B
JCHAIN
MKI67
IgA
IgG
IgG1
IgG2
IgG3
IgM
NA

B

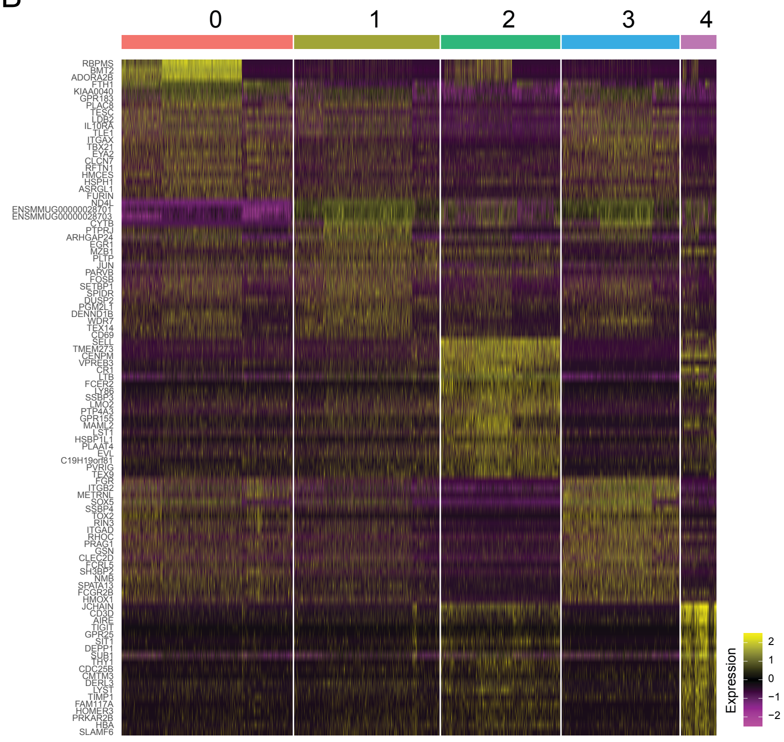

0
1
2
3
4
Expression

Figure S7

Table S1. Rabbit study schema. All animals were immunized at weeks 0, 8, and 24.

Group	N	Immunogen	mRNA/Protein	Dose (μg)	Route	Adjuvant
1	6	BG505 MD39.2 gp140	mRNA	100	IM	none
2	6	BG505 MD39.3 gp140	mRNA	100	IM	none
3	6	BG505 MD39.2 gp151	mRNA	100	IM	none
4	6	BG505 MD39.3 gp151	mRNA	100	IM	none
5	6	BG505 MD39.3 CD4KO gp151	mRNA	100	IM	none
6	6	BG505 gp120 foldon	Protein	30	IM	SMNP (375 μg)
7	6	BG505 MD39.3 soluble trimer	Protein	30	IM	SMNP (375 μg)
8	6	BG505 MD39 soluble trimer	Protein	30	IM	SMNP (375 μg)

Table S2. Flow Panel for B_{Mem} analysis

Antibodies	Clone	Source	Cat #
Streptavidin BV711 (empty)	-	BioLegend	405241
Streptavidin AF647 (MD39.3)	-	BioLegend	405237
Streptavidin BV421 (MD39.3)	-	BioLegend	405225
Streptavidin PE (MD39.3 bKO)	-	BioLegend	405245
Fixable Viability Dye eFluor506	-	Thermo Fisher Scientific	65-0866-18
Mouse anti-human CD4 BV650	OKT4	BioLegend	317436
Mouse anti-human CD8a APC-eFluor780	RPA-T8	Thermo Fisher Scientific	47-0088-42
Mouse anti-human CD16 APC-eFluor780	CB16	Thermo Fisher Scientific	47-0168-42
Mouse anti-human CD20 AF700	2H7	BioLegend	302322
Mouse anti-human IgG BUV737	G18-145	BD Biosciences	612819
Mouse anti-human CD27 PE-Cy7	O323	Thermo Fisher Scientific	25-0279-42
Mouse anti-human CD3 BUV395	SP34-2	BD Biosciences	564117
Goat anti-human IgD AF488	polyclonal	SouthernBiotech	2030-30
Mouse anti-human IgM PerCP-Cy5.5	G20-127	BD Biosciences	561285

Table S3. Flow Panel for B_{GC} analysis

Antibodies	Clone	Source	Cat #
Streptavidin AF647 (MD39.3)	-	BioLegend	405237
Streptavidin BV421 (MD39.3)	-	BioLegend	405225
Streptavidin PE (MD39.3 bKO)	-	BioLegend	405245
Fixable Viability Dye eFluor506	-	Thermo Fisher Scientific	65-0866-18
Mouse anti-human CD4 BV711	OKT4	BioLegend	317440
Mouse anti-human CD8a APC-eFluor780	RPA-T8	Thermo Fisher Scientific	47-0088-42
Mouse anti-human CD16 APC-eFluor780	CB16	Thermo Fisher Scientific	47-0168-42
Mouse anti-human CD20 AF488	2H7	BioLegend	302316
Mouse anti-human IgG BUV737	G18-145	BD Biosciences	612819
Mouse anti-human CXCR5 PE-Cy7	Mu5UBEE	Thermo Fisher Scientific	25-9185-42
Mouse anti-human CD3 BUV395	SP34-2	BD Biosciences	564117
Mouse anti-rhesus CD38 PE-Cy5	OKT10	In house	-
Mouse anti-human IgM PerCP-Cy5.5	G20-127	BD Biosciences	561285
Mouse anti-human PD1 BV605	EH12.2H7	BioLegend	329924
Mouse anti-human CD71 PE-CF594	L01.1	BD Biosciences	custom

Table S4. Flow Panel for sorting of MD39.3-specific B_{Mem} cells

Antibodies	Clone	Source	Cat #
Streptavidin AF647 (MD39.3)	-	BioLegend	405237
TotalSeq-C Streptavidin BV421 (MD39.3)	-	BioLegend	custom
TotalSeq-C Streptavidin PE (MD39.3 bKO)	-	BioLegend	405155
Fixable Viability Dye eFluor506	-	Thermo Fisher Scientific	65-0866-18
Mouse anti-human CD8a APC-eFluor780	RPA-T8	Thermo Fisher Scientific	47-0088-42
Mouse anti-human CD16 APC-eFluor780	CB16	Thermo Fisher Scientific	47-0168-42
Mouse anti-human CD14 APC-Cy7	M5E2	BioLegend	301820
Mouse anti-human CD3 APC-Cy7	SP34-2	BD Biosciences	557757
Mouse anti-human CD20 BUV395	2H7	BD Biosciences	563781
Mouse anti-human IgG BV605	G18-145	BD Biosciences	563246
Mouse anti-human CD27 PE-Cy7	O323	Thermo Fisher Scientific	25-0279-42
Goat anti-human IgD AF488	polyclonal	SouthernBiotech	2030-30
Mouse anti-human IgM PerCP-Cy5.5	G20-127	BD Biosciences	561285

Table S5. Flow cytometry AIM and ICS panel staining for MD39.3-specific T cells

Antibodies	Clone	Source	Cat #
LIVE/DEAD Fixable Blue	-	Invitrogen	L23105
GolgiPlug	-	BD Biosciences	555029
GolgiStop	-	BD Biosciences	554724
Mouse anti-human CD40	HB14	Miltenyi	130-094-133
Mouse anti-human CXCR5 PE-Cy7	MU5UBEE	Thermo Fisher Scientific	25-9185-42
Mouse anti-human CCR7 BV650	G043H7	BioLegend	353233
Mouse anti-human CD69 PE-Cy5	FN50	BioLegend	310908
Mouse anti-human CD137 (4-1BB) BV421	4B4-1	BioLegend	309819
Mouse anti-human CD25 BV605	BC96	BioLegend	302631
Mouse anti-human CD40L BB515	24-31	BD Biosciences	568170
Mouse anti-human CD134 (OX40) PE	L106	BD Biosciences	340420
Mouse anti-human CD8 BUV496	RPA-T8	BD Biosciences	612943
Mouse anti-human CD14 APC-Cy7	M5E2	BioLegend	301820
Mouse anti-human CD16 APC-eFluor780	eBioCB16	Thermo Fisher Scientific	47-0168-42
Mouse anti-human CD20 APC-Cy7	2H7	BioLegend	302314
Mouse anti-human CD3 BUV395	SP34-2	BD Biosciences	564117
Mouse anti-human CD4 PerCP-Cy5.5	OKT4	BioLegend	317428
Mouse anti-human PD-1 BV785	EH12.2H7	BioLegend	329929
Mouse anti-human CD45RA PE-CF594	5H9	BD Biosciences	565419
Armenian Hamster anti-ICOS BV480	C398.4A	BD Biosciences	566087
Mouse anti-human IFN-γ BUV737	4S.B3	BD Biosciences	612845
Rat anti-human IL-2 BV750	MQ1-17H12	BD Biosciences	566361
